# Persistence of poliovirus types 2 and 3 in waste-impacted water and sediment

**DOI:** 10.1371/journal.pone.0262761

**Published:** 2022-01-26

**Authors:** Allison Kline, Kara Dean, Alexandra L. Kossik, Joanna Ciol Harrison, James D. Januch, Nicola K. Beck, Nicolette A. Zhou, Jeffry H. Shirai, David S. Boyle, Jade Mitchell, John Scott Meschke

**Affiliations:** 1 Department of Environmental and Occupational Health Sciences, School of Public Health, University of Washington, Seattle, WA, United States of America; 2 Biosystems & Agricultural Engineering, Michigan State University, East Lansing, Michigan, United States of America; 3 PATH, Seattle, WA, United States of America; Institut Pasteur, FRANCE

## Abstract

Eradication of poliovirus (PV) is a global public health priority, and as clinical cases decrease, the role of environmental surveillance becomes more important. Persistence of PV and the environmental factors that influence it (such as temperature and sample type) are an important part of understanding and interpreting positive environmental surveillance samples. The objective of this study was to evaluate the persistence of poliovirus type 2 (PV2) and type 3 (PV3) in wastewater and sediment. Microcosms containing either 1) influent wastewater or 2) influent wastewater with a sediment matrix were seeded with either PV2 or PV3, and stored for up to 126 days at three temperatures (4°C, room temperature [RT], and 30°C). Active PV in the liquid of (1), and the sediment and liquid portions of (2) were sampled and quantified at up to 10 time points via plaque assay and RT-qPCR. A suite of 17 models were tested for best fit to characterize decay of PV2 and PV3 over time and determine the time points at which >90% (T90) and >99% (T99) reduction was reached. Linear models assessed the influence of experimental factors (matrix, temperature, virus type and method of detection) on the predicted T90 and T99 values. Results showed that when T90 was the dependent variable, virus type, matrix, and temperature significantly affected decay, and there was a clear interaction between the sediment matrix and temperature. When T99 was the dependent variable, only temperature and matrix type significantly influenced the decay metric. This study characterizes the persistence of both active and molecular PV2 and PV3 in relevant environmental conditions, and demonstrates that temperature and sediment both play important roles in PV viability. As eradication nears and clinical cases decrease, environmental surveillance and knowledge of PV persistence will play a key role in understanding the silent circulation in endemic countries.

## Introduction

Eradication of poliovirus (PV) has been a global public health priority since 1988, when the World Health Assembly declared eradication by 2000 a goal, and with the subsequent creation of the Global Polio Eradication Initiative (GPEI) [1]. PV eradication is within reach given the recent declaration of Nigeria as free from wild poliovirus on 25 August 2020, as only two countries remain endemic for wild PV: Afghanistan and Pakistan [2]. Strategies the GPEI employed with vaccination to accomplish the decrease in cases over time include Acute Flaccid Paralysis (AFP) surveillance, which follows cases of paralysis, and environmental surveillance (ES), which targets testing of water and sewage samples to determine areas of PV transmission [3–5]. Up to 90% of people infected with PV are asymptomatic with only approximately 1% of cases developing paralysis symptoms [6]. Due to this, as clinical cases of poliomyelitis decrease, PV ES becomes an increasingly important tool to detect the silent circulation of PV [7–11].

One limitation of PV ES previously discussed is that it can only measure PV presence at a singular point in time [12]. Thus, PV ES is unable to confirm whether a positive PV sample originates from recent transmission or from circulation in the environment due to the persistence of the virus. Studies following the withdrawal of the oral polio vaccine (OPV) or time after OPV vaccination campaigns found a decrease in Sabin-like (SL) PV over a few months [13,14], and sequencing results indicated that PV detected greater than four months after the switch from OPV to IPV (inactivated poliovirus vaccine) were due to importation [14,15]. The more recent global switch in April 2016 from the use of trivalent OPV (tOPV) to bivalent OPV (bOPV) with the removal of Sabin PV type 2 from the OPV due to concerns over vaccine-derived PV type 2 (VDPV2) has seen increased VDPV2 outbreaks and consistent positive ES samples [16–18]. This large number of outbreaks of circulating VDPV2 and frequent detections in PV ES samples following the vaccine switch raises questions regarding the persistence of the virus in the environment.

Understanding the persistence of PV in the environment and the environmental factors that influence persistence such as temperature, and water or sediment type, is important to make the distinction between recent transmission and viral survival in positive environmental samples. Previous studies characterizing PV persistence and influential factors focused mainly on PV type 1 (PV1) in sterile environments [19–29]. Two studies have focused on PV1 in human waste, with one conducted in an estuarine environment containing secondary treated domestic wastewater [30], and the other study conducted with mixed human and swine waste [31]. The limits of these studies showed a need for recent, updated persistence studies using PV types 2 and 3 (PV2 and PV3) in waste-impacted and microbiologically active environments similar to what would be found in Pakistan and Afghanistan.

The gold standard for PV detection in concentrated PV ES samples is virus amplification by cell culture followed by intratypic differentiation (ITD) using reverse transcription polymerase chain reaction (RT-PCR) to identify viral type or strain, with genotyping then employed to support epidemiologic tracking [32–35]. There has been a desire to shift to RT-qPCR due to the rapid and specific results that can be obtained and of particular importance in cases of silent transmission or circulation [36,37]. However, molecular and culture assays can yield different results due to the issue of viability, with RT-qPCR detecting the target for a longer period of time [38,39]. Previous studies have used RT-qPCR to quantify persistence of PV and compare with cell culture methods [26–28], but these studies have the same limitations as others previously mentioned: they were completed using PV1, therefore, PV2 and PV3 data are lacking and they utilized different environmental media.

The objective of this study was to evaluate the persistence of PV2 and PV3 in microbiologically active wastewater and sediment at different temperatures (4°C, Room temperature [~21°C], and 30°C) using both cell culture (BGMK cells) and molecular methods (RT-qPCR) in order to inform PV ES activities focused on the detection of PV in endemic areas.

## Materials and methods

### Wastewater and sediment sources

Approximately 6 L of wastewater was collected at the wastewater influent division channel at a local Seattle wastewater treatment facility on 14 June 2018 [PV3], 28 June 2018 [PV2] and 7 November 2018 [PV2/PV3 second set of experiments] and stored (4°C) for 0 to 4 days until use. Sediment for all samples was collected from Alderwood, WA, USA and was a gravelly-sandy-loam. No permits were required for field site access, as sediments were collected from private property by the owner and not transported across state lines. Gravelly-sandy-loam was chosen due to its similarity to the textural composition of soil found in Pakistan where PV outbreaks still occur [40]. The sediment was mixed in a 1:1 ratio with waste solids obtained from a local Seattle wastewater treatment facility to simulate sediments in waste-impacted waters in Pakistan [41,42].

### Poliovirus

Sabin type 2 (P712 Ch 2ab) and Sabin type 3 (Leon 12a1b) vaccine strain PV stocks were provided by the United States Centers for Disease Control and Prevention (CDC) and were grown separately on Buffalo Green Monkey Kidney (BGMK) cells through confluent lysis of cell monolayers [43]. BGMK cells were chosen for use rather than L20B or RD cells, as quantification via plaque assay was desired over TCID50. Viral amplification prior to RT-qPCR was not needed due to the concentration of PV seeded and the desire for quantitative over qualitative data. Cultured stocks were frozen in cell culture cryogenic plastic tubes (Midland Scientific, Denver, CO, USA) at -80°C until use. PV2 and PV3 stocks were diluted 5-fold into a total of 10 mL of 1X Phosphate-buffered saline (PBS; pH 7.0) to achieve a concentration of ~5 x 105 plaque forming units [PFU]/mL.

### Microcosms

The relationship between PFU of PV2 and PV3, time [0–126 d], temperature [4 oC, RT, 30 oC], and type of sample (wastewater or sediment) was tested using a longitudinal experimental study with controlled microcosms. Room temperature varied between 19.6°C and 23°C, and was determined by a temperature probe that recorded measurements every 10 minutes.

The temperatures chosen for this experiment [4 oC, RT, 30 oC] encompass the range of temperatures seen in the Indus River Basin which covers most Pakistan with the max temperature 30 oC being chosen more conservatively as a high temperature due to the rapid nature of PV decline at high temperatures. The Upper Indus Basin has average minimum and maximum temperatures of ~4 oC and 13 oC in the winter months and ~19 oC and 32 oC in the summer months. The Lower Indus basin has average minimum and maximum temperatures of ~10 oC and 24 oC in the winter months and ~25 oC and 36 oC in the summer months [44].

**Fig 1:**
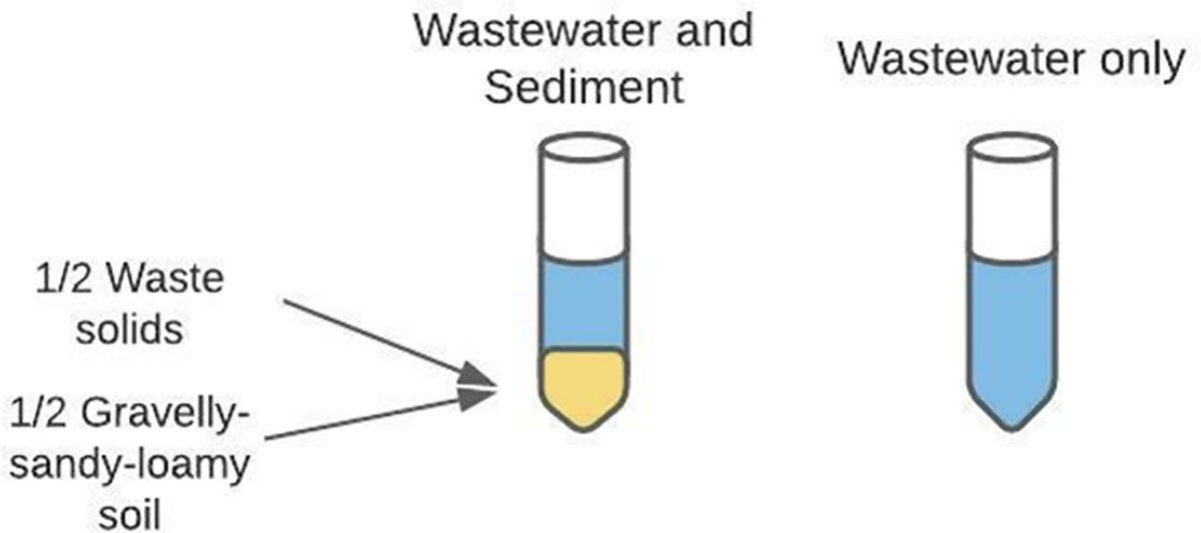
Microcosms evaluated in current study. Figure created using Microsoft PowerPoint.

Two different microcosm types were prepared with three different sample types: 1) WWO, wastewater only, comprised of domestic influent wastewater only to test for PV persistence in the absence of sediment, and 2) MM, mixed microcosms, with both domestic wastewater and a sediment microcosm (1:1 ratio by mass of waste biosolids and gravelly-loamy-sand) (Fig 1). When processed for analysis, MM were split into two sample types Sediment and Wastewater (MM-S and MM-W respectively). Microcosms were prepared in sterile conical 50-mL centrifuge tubes [VWR; Radnor, PA, USA] with each tube a time point and temperature so that wastewater and sediment could be separated easily through direct centrifugation, as described previously [21,45]. Both waste solids and soil were used as wet weight to emulate environmental conditions. MM tubes had 3 g of sediment to 10 mL of wastewater. WWO microcosms contained 10 mL of wastewater. The MM tubes were prepared in duplicate for each time point and temperature yielding 60 MM microcosm tubes per virus (2 duplicates x 10 time points x 3 temperatures). The WWO tubes were prepared singularly for each time point and temperature yielding 30 WWO microcosm tubes per virus (10 time points x 3 temperatures). The experiment was performed separately each for PV2 and PV3. A second, smaller scale duplicate experiment with both MM and WWO microcosms was performed with three time points (0, 21, 84 days) each for PV2 and PV3 to confirm repeatability of results.

All microcosms per experiment were seeded individually to ensure even distribution between samples with 500 µL of the stock dilution and vortexed for 5 min to evenly distribute the virus within the sample. All samples were then settled (24 h, RT) in order to ensure separation between sediment and wastewater portions prior to processing the first time point. After 24 h, 30 tubes were incubated at room temperature, 30 tubes refrigerated at 4°C, and 30 tubes incubated at 30°C. At day 0 samples were processed for initial quantification of PFU; 1 MM-S sample, 1 MM-W sample, and 1 WWO sample representing each temperature were incubated for 1 h at their respective temperatures (4°C, RT or 30°C). Additional sample sets were then processed at days 1, 3, 7, 14, 21, 28, 42, 84, and 126.

### Processing sediment from MM microcosms

For extraction of PV2 and PV3 from sediments for downstream detection and quantification, a method used to isolate PV from oyster tissue was adapted for use in waste-impacted sediment [46,47]. The MM samples were centrifuged (5,000 x g, 20 min) to separate wastewater and sediment. The supernatant, which is the wastewater portion (10 mL), was pipetted off and retained for separate processing (see *Processing wastewater from MM and WWO samples* section below). The sediments were processed after the wastewater portion was removed using a 2M NaNO3 in 3% beef extract eluent at a 3:1 (W/V) ratio, vortexed (5 min), and shaken (15 min) to dissociate the viral particles from the sediment. Samples were then centrifuged (5,000 x g, 20 min) and the supernatant removed for processing. A 3% sterile skim milk solution (Oxoid, Ltd., Hants, UK) was added at a 0.1% volume concentration, the pH modified with 5M HCl between 3 and 4 using pH strips, and samples shaken (2 h) to flocculate. After shaking, samples were then centrifuged (3,500 x g, 30 min). The supernatant was removed, leaving the floc pellet containing the virus. PBS (5 mL) was added to re-suspend the virus [48]. A Vertrel ^TM^ XF [Miller-Stephenson, Danbury, CT, USA] extraction was utilized as a replacement for chloroform extraction to remove organics from the sample using the following protocol [49]: 0.5X volume of Vertrel XF was added to samples, samples were vortexed (2 min), centrifuged (3,000 x g, 15 min), and the supernatant was pipetted off carefully. The Vertrel-extracted samples were stored at 4ºC prior to quantification via plaque assay or -80ºC prior to quantification via RT-qPCR.

### Processing wastewater from MM and WWO samples

Ten (10) mL of the wastewater for both MM-W and WWO samples were placed in a new centrifuge tube for processing. One (1) mL of each sample was stored at -80°C for use in RT-qPCR. A Vertrel XF extraction was completed on the remaining 9 mL to remove organics as previously described [48,49]. Then a skim milk secondary concentration step was performed as described in the sediment processing step above. The Vertrel-extracted samples were stored at 4ºC prior to quantification via plaque assay.

### Plaque assay

BGMK cells were maintained in Minimum Essential Medium Eagle (MEM) [Corning, Corning, NY, USA], with 10% Fetal Bovine Serum (FBS) [ATCC; Manassas, VA, USA] in 25-cm2 polystyrene flasks with vented caps [Corning; Corning, New York, USA] and incubated (37°C). Prior to infection, cells were transferred into 9.5-cm^2^, 6-well tissue culture plates[Corning; Corning, New York, USA] seeded at ~6 x 10^4^ cells and grown for 4 days to 95% confluent monolayers before inoculation with the sample [50,51]. Ten-fold dilution series in PBS were prepared with the MM-W, MM-S and WWO processed samples stored at 4°C, with time points farther out requiring less dilution. Each sample had three dilutions which were chosen based on the time point to ensure that it was detectable. For each dilution, a volume of 200 µL was added to each well in triplicate and incubated for 1 h, swirling every 15 min to ensure virus absorption and even distribution of virus onto the cells. Cells were overlaid with ~2.5 mL of an Avicel overlay [FMC Health and Nutrition, Philadelphia, PA, USA] [52], which contained filter-sterilized L-glutamine at a final concentration of 2 mM [Corning, Corning, NY, USA], 0.356% HEPES [Fisher Bioreagents; Pittsburgh, PA, USA], 0.01% gentamicin sulfate [Sigma-Aldrich], 0.01% kanamycin sulfate [Sigma-Aldrich], 0.225% sodium bicarbonate [Sigma-Aldrich], 1.2% Minimum Essential Medium Eagle (MEM) [Sigma-Aldrich], 1.2% nystatin [Sigma-Aldrich], 1.5% sterile Avicel in de-ionized water, and 2% FBS [52]. The infected BGMK cells were incubated (40–44 hours, 37°C, 5% CO2), and then stained with 2% crystal violet in 20% methanol. Plaques were counted and the PV2 or PV3 PFU per sample was determined. The starting level in the microcosms ranged from 10^3.99^ to 10^5.37^ PFU PV2 and PV3 ranged from 10^2.00^ to 10^4.68^ PFU PV3, which is similar to what has been seen in raw sewage [53,54], and levels at the end were similar to what is seen in the environment and varies based on conditions (10^1.26^ to 10^4.67^ PFU PV2 and undetected to 10^3.95^ PFU PV3).

### Viral RNA extraction and qPCR

Samples stored at -80°C for downstream RT-qPCR analysis were extracted using the vacuum extraction method for the QIAamp Viral RNA Mini Kit, [Cat No./ID: 52906, QIAGEN, Germantown, MD, USA], with an input volume of 140 µL of sample and elution volume of 60 µL. RNA was stored at -80°C.

After viral extraction, RT-qPCR was performed using QuantaBio qScript XLT One-Step RT-qPCR ToughMix (10 µL/reaction) containing AccuStart II Taq DNA Polymerase and nuclease-free water (3.8 µL/reaction) along with assay-specific forward and reverse primers (10 µM concentration, 0.5 µL/reaction each), probe (10 µM concentration, 0.2 µL/reaction), and sample RNA (5 µL/reaction). Reactions were performed using the Bio-Rad CFX96 Deep Well Real-Time System [Bio-Rad Laboratories, Hercules, CA, USA]. PV2 and PV3 primers and probes used are described in Nijst et al and are displayed in 5’ to 3’ orientation [55], (PV2 Sabin type 2 forward primer AAGGAATTGGTGACATGATTGAGG, Sabin type 2 reverse primer CTCGGCTTTGTGTCAGGC, Sabin type 2 probe FAM-TGGAAGTCGGGGGAACCAATGC-BHQ1, PV3 Sabin type 3 forward primer AATGACCAGATTGGTGATTCCTTG, Sabin type 3 reverse primer GTAAATGCGGACTTTGGAGGTTACT, Sabin type 3 probe FAM-TGTGATCATTGACAACACGAACTGCCAA-BHQ1). These primers and probes were chosen for use rather than the ITD 5.0 RT-PCR methods [34] as the ITD primers and probes are designed to be degenerated to allow for broad PV detection and are used following viral amplification and so viral RNA was detected and not virus isolated in cells. The assay sensitivity was critical in this study. As the samples processed in this study are directly detected rather than amplified via cell culture, the improved specificity and improved or similar sensitivity (previously reported as PV2: 100 CCID50/mL [55] vs. 10,000 CCID50/mL [34] and PV3: 1,000 CCID50/mL by both methods [34,55]) was critical. The reaction for PV2 was performed at 50°C for 45 minutes, 95°C for 15 minutes followed by 45 cycles of 95°C for 5 seconds, 58°C for 15 seconds, and 72°C for 5 seconds, and ending with 30 seconds at 40°C. The reaction for PV3 was performed at 50°C for 45 minutes, 95°C for 15 minutes followed by 45 cycles of 95°C for 5 seconds, 56°C for 15 seconds, and 72°C for 5 seconds, and ending with 30 seconds at 40°C. Ten-fold dilutions of the sample and no template controls were routinely run. A Cq cutoff for RT-qPCR samples was set at 40 Cq. RT-qPCR and plaque assay data were compared using curves generated through the analysis of PV2 and PV3 virus stocks where titers were analyzed by both RT-qPCR and cell culture. These standard curves were then used to convert all other RT-qPCR Cq values to the cell culture equivalent in PFU, so as to enable comparisons across datasets (S1 and S2 Tables).

### Statistical analyses

Accounting for each of the two viruses, three temperatures, three sample types and both culture and molecular data, a total of 35 experiments were performed and analyzed (due to an equipment malfunction, RNA extracts for one set were lost). A suite of 17 different one-, two-, and three-parameter models were fit to each set of the experimental results using Maximum Likelihood Estimation methods as extensively described in prior works [56–59] and previously used to model viral persistence: exponential [60], logistic [61], Fermi [58,62], exponential damped [63], Juneja and Marks 1 [64], Juneja and Marks 2 [65], Gompertz-Makeham [66], Gompertz 2 [67], Gompertz 3 [68], Weibull [69], lognormal [70], gamma [71], broken-line [72], broken-line 2 [72], double exponential [73], sigmoid type A [74], and sigmoid type B [74]. The log(PFU) values from the plaque assays and PFU calculated values from the RT-qPCR assays were set as the dependent variables with time in days defined as the independent variable. For this analysis, each experiment and all of its replicates were considered one dataset to account for measurement error. The statistical programming language R, version 3.6.2, was used for model fitting and the subsequent analyses with *optim* and *mle* functions in the stats and stats4 packages [75]. Before the 17 models were fit to the data, each dataset was tested for a significant trend of decay. If no significant decline was observed the dataset was excluded from the model fitting. The fit of each model was evaluated with visual inspection and three metrics: the Bayesian Information Criterion (BIC) as a means of model comparison metric; the normalized root mean square error (nRMSE) and adjusted R^2^ values as indicators of goodness of fit to the data. The lowest BIC indicates the best fitting model of those fit; however, models with BIC values that differ by less than two BIC units are considered equivalent in terms of selection [76]. The RMSE for each model was normalized by dividing by the range of observed log reductions, as shown in Eq 1, where *LR*_*P*_ is the predicted log reduction value, *LR*_*O*_ is observed log reduction value, a *N* is the number of time points. The RMSE value is an absolute measure of fit, with an RMSE of 0 indicating a perfect fit. Normalizing the RMSE allows for a more direct comparison between the datasets tested. Given the high degree of variability inherent in microbial data, a nRMSE<0.20 was considered indicative of a good fit as there is no standard threshold and values should be evaluated within specific datasets and models. Adjusted R^2^ values were also evaluated to confirm goodness of fit as it provides a commonly used and widely recognized metric. However, it should be noted that nonlinear data sets are expected to have lower R^2^ values than linear models (<< 1).


$$nRMSE=\frac{\sqrt{\frac{\sum {\left({LR}_{P}-{LR}_{O}\right)}^{2}}{N}}}{{max(LR}_{O})-min({LR}_{O)}}$$ (1)

The best fitting models for each dataset were used to calculate the time points at which >90% (T90) and >99% (T99) reduction were reached. Calculated T90s/T99s were only reported for datasets that had a model providing a good fit to the data or that observed a 1-log/2-log reduction during the experimental period. All of the calculated T90/T99s were used in the factor analysis, as they were still considered representative of general high or low T90/T99 values. To perform the factor analysis, a linear model (Eq 2) was specified to assess the influence of the different experimental factors, independent variables—Virus type, Temperature, Matrix, and Method of detection—with dependent variables T90 or T99. As shown in Eq 2, for T90, *β*_0_ is the linear model intercept and *β_i_* are the coefficients for the experimental variables *x_i_*.


$$T90=\beta_{0}+\sum_{i=1}^{n}\beta_{i}x_{i}$$ (2)

Before fitting Eq 2 using the *lm* function in R, interaction plots were evaluated to assess the interactions between experimental factors [75]. Dummy variables were established to represent levels of the following categorical variables: Virus type, Matrix, and Method of detection, while Temperature and Time (T90 or T99) were treated as continuous variables. The reference condition, represented by the intercept in Eq 1, was based on the selection of the dummy variables. In the analysis, PV3, WWO, RT-qPCR, and 4°C were set as the reference conditions. An ANOVA in R was used to determine if the more complex models (that included interaction terms) provided a significantly better fit to the data than the model with only main effects for each experimental variable [75]. If the interaction terms did not significantly improve the model, they were removed from the analysis to avoid overfitting and conserve degrees of freedom.

## Results

### Persistence model fitting

**Fig 2:**
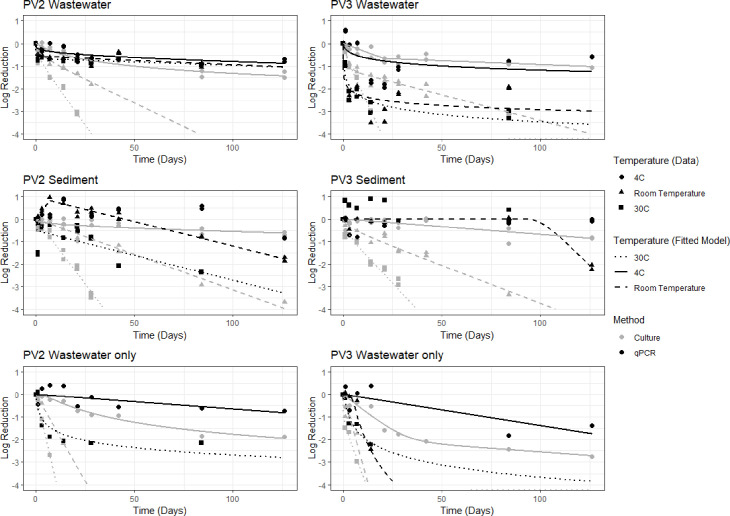
The best fitting model for each dataset separated by virus type and matrix. The data from culture experiments and the best fitting models to culture datasets are shown with grey points and grey lines. The data from qPCR experiments and the best fitting models to qPCR datasets are shown with black points and black lines.

A best fitting model was determined for 32 of the 35 experiments. For experiments evaluated via RT-qPCR, no significant log reductions were observed in the data over time for the following samples: PV2 Sediment at 4°C, PV3 Sediment at 4°C, and PV3 Sediment at 30°C. The best fitting models for the 32 experiments are shown in Fig 2 (the best fitting model, optimized parameters, and goodness of fit metrics for each dataset are available in S3 Table). For the 32 experiments, 25 had a best fitting model with a nRMSE less than 20%. The seven remaining models with a nRMSE > 20% were also associated with adjusted R^2^ values of less than 0.60, further confirming the lack of fit of the model.

Using the best fitting models for each experiment, the time required to achieve 90% PFU reduction (T90) and 99% PFU reduction (T99) were calculated (Table 1). There is more uncertainty associated with the T90 and T99 values predicted for the seven datasets that did not have a model with a good fit to the data; however, for 28 of the 32 fitted experiments, a one-log reduction was observed within the 126-day experiment time period. A two-log reduction was observed for only 20 of the 32 fitted experiments within that timeframe. Because the T90 values were within or closer to the observed 126-day experiment period, there is less uncertainty associated with the T90 predicted values than the T99. Generally, there is more certainty in model predictions within a domain of applicability related to the empirical observations. T90s/T99s are only reported in Table 1 for the datasets that had a model providing a good fit to the data, or that observed a 1-log/2-log reduction during the experimental period.

**Table 1:** Predicted T90 and T99 values for PV2 and PV3 reduction in waste-impacted microcosms.

	Time (days)			
Matrix	Estimated time to 90% (99%) Cq reduction		Estimated time to 90% (99%) PFU reduction	
	PV2	PV3	PV2	PV3
4°C				
MM: Sediment	NA	NA	NP (NP)	NP (NP)
MM: Wastewater	NP (NP)	48 (NP)^a^	48 (395)	111 (332)
WWO: Wastewater only	NP (NP)	72 (NP)^a^	37 (137)	16 (36)
Room temperature (19°C–24°C)				
MM: Sediment	90 (138)	119 (125)	32 (63)	20 (49)
MM: Wastewater	113 (375)	0.1 (3)	11 (33)	4 (38)
WWO: Wastewater only	NA	7 (11)	6 (12)	3 (7)
30°C				
MM: Sediment	22 (67)^a^	NA	9 (17)	6 (16)
MM: Wastewater	106 (435)	1 (2)	4 (12)	4 (9)
WWO: Wastewater only	3 (23)	3 (12)	3 (5)	0.3 (3)

NP: Not Published because the best fitting model did not provide a good fit (nRMSE>0.20) and a 1-log (or 2-log) reduction was not observed during the experimental period.

^a^: Best fitting model did not provide a good fit (nRMSE > 0.20) but a 1-log (and/or 2-log) reduction was observed.

### Factor influence

Interaction plots between the experimental factors were evaluated before the linear models were fit. When T90 was the dependent variable of concern, Temperature and Matrix were the only two independent variables with a strong interaction. Although the same general trend of higher T90 values at 4°C and lower T90 values at 30°C was observed in all three matrices, the effect of Temperature was more significant for the Sediment matrix. When T99 was the dependent variable, the Temperature and Virus variables and the Method and Matrix variables had significant interactions. These interaction terms were incorporated into each model, and analysis of variance (ANOVA) assessed if their inclusion added significant value to the analysis.

**Fig 3:**
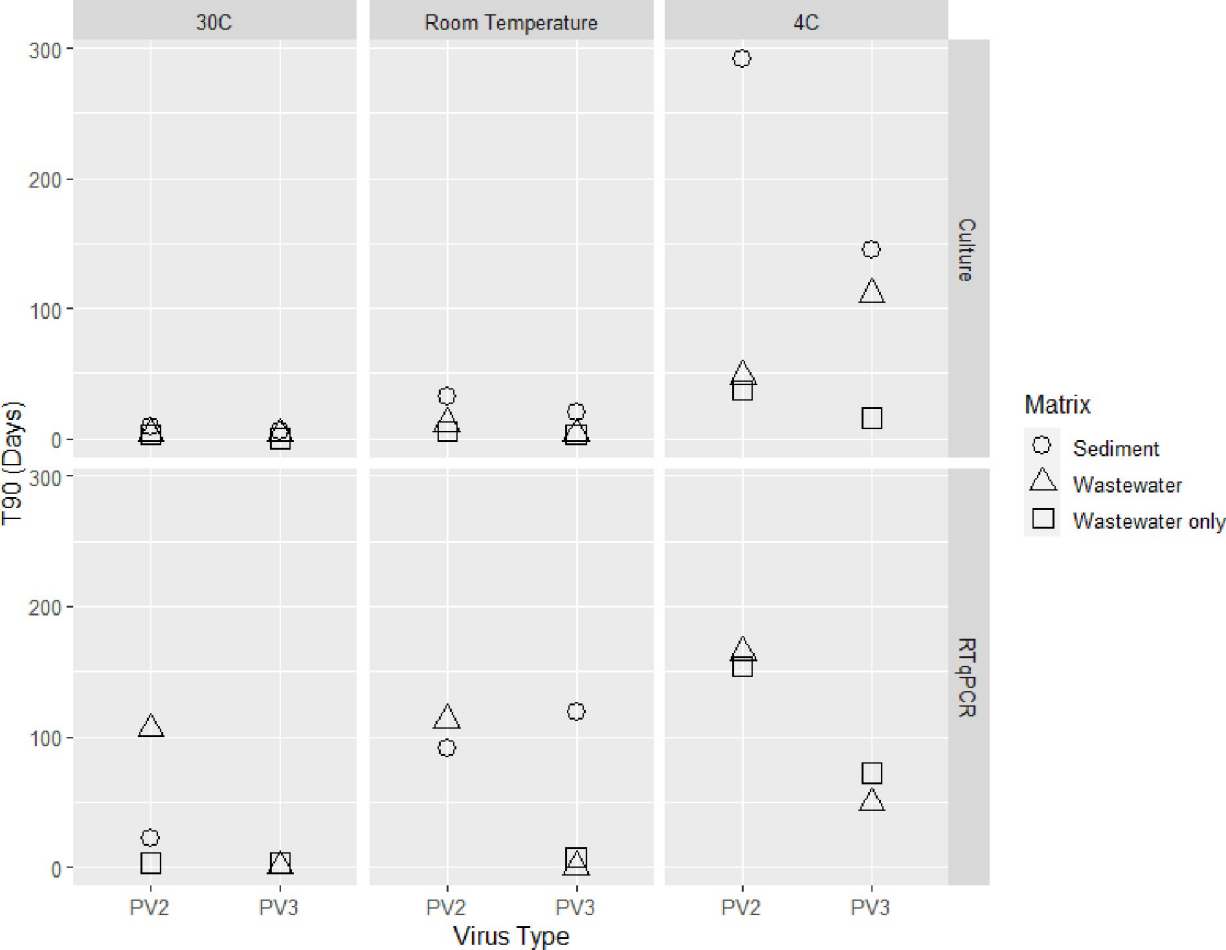
Visual presentation of the T90 values as influenced by the experiment matrix type, virus type, temperature, and method of detection. T90 is the time to 90% reduction. PV2 is poliovirus type 2. PV3 is poliovirus type 3.

When T90 was the dependent variable, the inclusion of the Temperature and Sediment interaction term significantly improved the model’s ability to capture the variability in the data. The model coefficients and significance values are shown in Table 2. The reference for the linear model was PV3 in the WWO matrix at 4°C with the RT-qPCR method, and the T90 associated with these conditions is represented by the intercept in Table 2. The PV2 and Sediment coefficients were significant and positive, the Temperature coefficient was significant and negative, and the Culture coefficient was mildly significant and negative. The Sediment and Temperature interaction term was also significant and negative. This is illustrated by the visual representation of the data shown in Fig 3 where the increase in temperature had a greater effect on decay in the Sediment matrices than in the Wastewater and WWO.

**Table 2:** Model coefficients for linear models of factor influence.

Dependent Variable: T90			Dependent Variable: T99		
	Estimate	Pr(>|t|)		Estimate	Pr(>|t|)
(Intercept)	70.5	6.49E-04	(Intercept)	402.4	5.80E-03
PV2	34.8	0.02	PV2	33.8	0.74
Wastewater	20.9	0.20	Wastewater	270.5	0.03
Sediment	159.9	1.39E-05	Sediment	170.2	0.21
Temperature	-2.6	1.20E-03	Temperature	-18.3	8.07E-04
Culture	-39.6	0.01	Culture	-177.9	0.10
Sediment:Temperature	-6.1	4.93E-04			
RSE	38		RSE	289	
DF	25		DF	26	
Multiple R^2^	0.75		Multiple R^2^	0.46	
Adjusted R^2^	0.69		Adjusted R^2^	0.35	

The categorial independent variables (Matrix, Method, and Virus Type) were assigned dummy variables for the analysis. The intercept for both the models in this table represent the reference conditions for the experiments: In this case, PV3 in Wastewater only at 4°C with qPCR methods. RSE is the residual standard error of the model, DF refers to the degrees of freedom in the model, and R^2^ is the model’s R-squared value.

Although there was less certainty associated with the T99 estimates, the factor analysis was also completed with the T99s as the dependent variable as this is more valuable information when considering the effect of persistence on a positive PV ES sample particularly as eradication of PV nears. The results of this analysis are also shown in Table 2. For the linear model with T99 as the dependent variable, no interaction terms significantly improved the fit of the model. (analogous visual representation for T99s available in S1 Fig). The reference for the model was PV3 in WWO at 4°C with RT-qPCR methods as in the previous analysis. The results of this analysis indicated that an increase in temperature increased decay as expected. However, Sediment was not a significant coefficient for this model, and instead Wastewater was positive and significant, suggesting that the Wastewater matrix would have higher T99 values than the WWO.

## Discussion

This study aimed to address gaps in the current literature with respect to ES of PV and the role of PV persistence in endemic areas and additionally address assumptions and limitations of current surveillance strategies when interpreting positive PV ES samples. First, since persistence of PV in sediment and water is largely dependent on soil type and water type [21–23,25,26,28,77], this study targeted a sediment type and microbiologically active wastewater conditions similar to what would be seen in endemic areas, specifically Pakistan, within the limitations of the study. Second, with the majority of PV persistence studies focusing on PV1, this study characterized persistence of PV2 and PV3 and assessed potential differences, particularly needed in light of recent VDPV2 outbreaks globally following removal of Sabin PV type 2 from OPV. Additionally, because a majority of the previous PV persistence studies were published more than 15 years ago, comparisons between RNA persistence and culturable virus survival are rare. To address the potential for using molecular methods exclusively in ES, this study compared decay of RNA and culturable virus. Characterizing the persistence of PV with respect to these factors allows for more accurate interpretations of PV ES samples in conjunction with AFP surveillance and demonstrates the need to consider persistence of PV in these interpretations.

A suite of 17 different persistence models were fit to the data in an effort to accurately fit the complex decay patterns observed including shoulders and long tails. The seven datasets that did not have a model providing a good fit to the data were predominantly RT-qPCR datasets. The RT-qPCR experiments resulted in a large amount of variability in recovered concentrations and this affected the model fitting. Four out of the seven datasets were PV2 data and three were PV3 data. Six of the seven datasets were from the 4°C trials, the lowest temperature used in these experiments. Lower temperatures are associated with greater persistence, and the lack of clear decay patterns in the 4°C experiments is reflected by the poor fit statistics (nRMSE>0.20) for these datasets.

The T90 and T99 values displayed in Table 1 and the corresponding visual of the models (Fig 2) suggest the potential for PV to persist for multiple months depending on the sample type and temperature similar to previous studies [20,21,23]. There is more confidence in the T90s compared to the T99s due to the T90s being closer to or within the experimental period of 126 days, yet both were used for interpretation of results. Estimated T99s at 4°C (Table 1) indicate the impact persistence could have on positive PV ES samples at low temperatures with PV projected to persist around half a year (PV2 WWO) to around a year (PV2 and PV3 MM-W). Even samples at Room temperature show potential for PV to persist around a month or two depending on sample type (Table 1), which may be important in evaluation of positive PV ES samples. At the highest temperature tested in this study, 30°C, estimated T99s display PV persistence of a few days to approximately two weeks with the shortest persistence in WWO microcosms (Table 1), demonstrating PV persistence was clearly dependent on sample type and temperature. Among all factors influencing decay of PV over time in this study, Temperature was consistently highly significant and negative (Table 2). This was consistent with previous studies on PV persistence and persistence of other viruses in environmental media (previously studied examples include echoviruses, rotaviruses and other enteroviruses such as coxsackieviruses) [19–21,25,78,79]. All are consistent with this study in that lower temperatures are protective of virus infectivity and higher temperatures lead to increased rates of decay. The interaction between Sediment and Temperature for the T90 estimates was particularly strong in this study being significant and negative (Fig 3, Table 2). The presence of Sediment itself was also important in this study with a positive and significant coefficient for the T90 estimate (Table 2) consistent with observed values. The T90 estimates of PV2 and PV3 reduction over time in the Sediment matrices had higher values at their respective temperatures when compared with the MM-W and WWO samples (Table 1, Fig 3). Comparisons of T90 and T99 values between the wastewater and WWO samples further demonstrate the protective effect of the sediment (Table 1); this is also shown by the significant and positive Wastewater coefficient for the T99 estimates (Table 2). It has been previously hypothesized that sediment particles protect the viral capsid when attached [20,24,26,30,78].

The sediment type itself is also important in interpreting these results. The soil type and water type were chosen specifically in an attempt to emulate environmental conditions in Pakistan. A gravelly-sandy-loam was selected for use in this study for its similarity to soil types in Pakistan near areas where PV ES sampling frequently takes place [17]. This was critical as the specific soil type has been shown previously to play a role in persistence of viruses [26,77]. Sandy soil types have lower adsorption of PV and soils with more clay have higher adsorption, which is correlated with virus survival [26,77]. Additionally, microbial antagonism and the effect of sterile vs non-sterile environments has also been found to be important in PV persistence [22,23,26]. Previous research demonstrated that with increased filtration or sterilization of water, decay rates of PV1 are slower [22,23,26]. Therefore, the use of wastewater and biosolids in this study were important to make the results applicable to endemic areas.

The length of PV persistence and the role of sediment and temperature found in this study are important in the context of ES. PV persistence is not included in models or risk analyses in the current literature that use PV ES in modelling approaches [80,81], and should be considered in the interpretation of PV ES results and subsequent decision making. Currently, in Pakistan, ES samples are collected and processed monthly for PV presence differentiated by type from 75 sites across the country in addition to AFP surveillance when cases arise [17]. As ES samples are only collected monthly, PV persistence of multiple months at 4°C (Table 1) and multiple weeks at higher temperatures (Table 1), could potentially confound a determination that a positive PV ES sample indicates the occurrence of active shedding. Thus, a better understanding of PV persistence and quantitative measures to observe declining trends could provide greater confidence in the interpretation of PV ES results.

When comparing to previous studies using PV1, the PV2 and PV3 survival results from this study are similar. In a persistence study done by Hurst [78] with PV1 using sandy-loamy soil in anaerobic, non-sterile conditions, T90 culturable PV1 was reached by 33 days at 23°C and 3.5 days at 37°C. This is comparable to this study in which gravelly sandy loam sediment samples (MM-S) in non-sterile conditions had estimated T90s for PV2 and PV3 at Room temperature of 32 and 20 days respectively and T90s for PV2 and PV3 at 30°C of 9 and 6 days respectively (Table 1). In the same study by Hurst using PV1, T90 culturable PV was not reached by 75 days at 1°C, with an estimated T90 of 323 days using linear regression. In this study, MM-S samples at 4°C did not reach a 1 or 2-log reduction within the 126 days of the study (Table 1). Since temperature has a large effect on PV persistence (Fig 3), the 3°C difference in the low temperature analysis (1°C in Hurst et al. vs 4°C in this study) may account for large difference seen in PV persistence between these studies. Additionally, because this study used a suite of 17 one-, two-, and three-parameter models to estimate decay over time (Fig 2) and the Hurst study used simple linear regression, this difference in analysis could impact the disparity in results particularly at the lower temperatures when estimation was outside the bounds of the study period. With the significant effects that soil type and temperature can have on persistence of PV in wastewater and sediment microcosms, direct comparisons of this study’s results to PV1 persistence studies can be difficult to interpret.

The method of analysis also influenced the decay of PV2 and PV3 with culturable virus via plaque assay having greater decay over time when compared to RT-qPCR, even at higher temperatures (Room temperature and 30°C) (Tables 1 and 2, Figs 2 and 3). The slower decay of PV2 and PV3 RNA compared to culturable virus in this study is consistent with previous research demonstrating the slower decay of PV1 RNA when compared to culturable virus [26–28]. This difference in decay suggests the importance of the continued use of culture data in PV ES to indicate areas with potential for outbreaks. Positive PV ES culture samples are confirmed through RT-qPCR to identify type or strain with the subsequent genotyping of positive isolates [5]. Even at high temperatures, the slow decay of RNA is important in interpretation of these results. Not considering persistence as a factor in interpretation of RT-qPCR results from positive PV ES samples could drastically impact any decisions made based on results from these samples if it was assumed to be from new cases arising in a region.

This study had a few limitations. As importation of sediment or wastewater samples directly from Pakistan was impractical, locally sourced sediment and wastewater was used as a proxy but these are not directly representative of all PV ES sample sites in Pakistan where this study’s results were focused. Future studies in Pakistan could confirm present findings with small-scale testing of ES samples. Additionally, the constraint of 3 temperatures and 10 time points tested limited the predictive ability of the models, particularly for RT-qPCR results and 4°C culture results that had PV persistence beyond the time points sampled. Additional time points throughout the study or later time points could have improved this limitation. Due to an equipment malfunction, 32 extracted RNA samples were lost and therefore could not be included in the RT-qPCR assays and subsequent analysis. This decreased the robustness of the RT-qPCR data as there were fewer time points compared to the culture data and resulted in increased uncertainty in the RT-qPCR modeled results. Finally, there is inherent variability in collecting, processing, and analysis of biological samples, which may contribute to the initial increase in PV2 and PV3 measurements in MM-S samples. Disaggregation of clumping viruses may have also contributed to the initial increase in the MM-S samples.

## Conclusion

This study’s characterization of PV2 and PV3 persistence suggests the sizable impact persistence has in addressing limitations of ES. First, the large effect sediment and temperature has on the viability of PV in the environment shows the need for incorporating sample type and temperature in interpretation of positive PV ES samples. It was important in the study that characterized decay of PV was specific to environmental conditions in Pakistan, where PV is endemic, as this allows for a more accurate depiction of PV persistence in that area. PV RNA decayed slower when compared to active virions, which indicates the value of culture data. The length of persistence of both culturable virus and RNA of PV2 and PV3 cautions interpretations of presence of active PV, type, strain, and sequence of positive PV ES samples. Persistence could affect decisions made based on PV ES results if it is assumed that results are due to recent shedding. Incorporating persistence into future decisions or models based on PV ES results will help address this gap. Future work should evaluate persistence of PV2 and PV3 in distinct sediment types for longer periods of time for cooler temperatures and focus on assessing persistence as PFU nears zero to fully characterize die-off of PV in the environment. As eradication nears and clinical cases decrease, and with the recent re-emergence of VDPV2 outbreaks, PV ES will play a key role in better understanding the silent circulation in endemic countries, with knowledge of PV persistence informing how best to interpret positive PV ES samples collected in those areas.

## Supporting information

S1 Fig Visual presentation of the T99 values as influenced by the experiment matrix type, virus type, temperature, and method of detection. T99 is the time to 99% reduction. PV2 is poliovirus type 2. PV3 is poliovirus type 3.
(DOCX)

S1 Table Experimental dataset. (XLSX)

S2 Table Formulas for Cq to PFU calculation. (XLSX)

S3 Table Best fitting models for each dataset. (DOCX)
