# Supplementary material for: Persistence of poliovirus types 2 and 3 in waste-impacted water and sediment
Source: PLoS One. 2022 Jan 26;17(1):e0262761. doi: 10.1371/journal.pone.0262761 (PMC8791527; doi:10.1371/journal.pone.0262761)
Supplement: S1 Fig — T99 is the time to 99% reduction. PV2 is poliovirus type 2. PV3 is poliovirus type 3. (DOCX) [file pone.0262761.s001.docx]

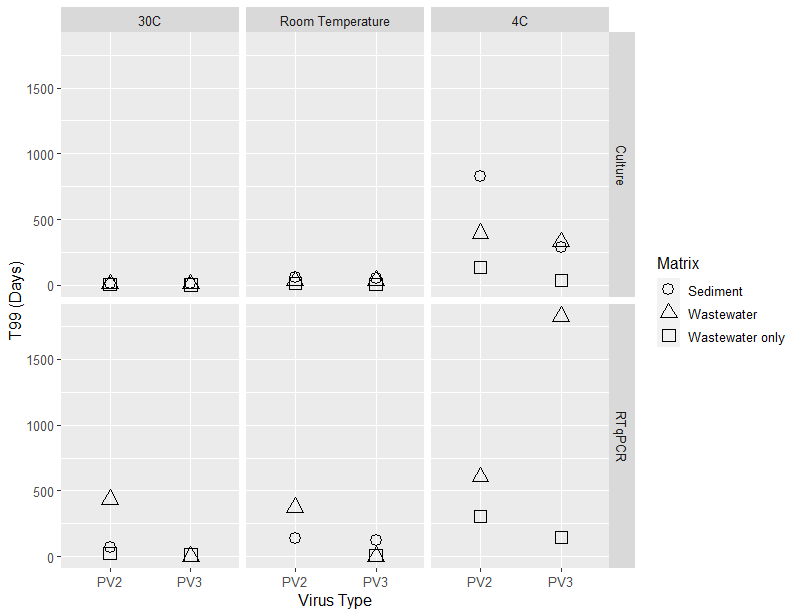


**Fig S1.** **Visual presentation of the T99 values as influenced by the experiment matrix type, virus type, temperature, and method of detection.** T99 is the time to 99% reduction. PV2 is poliovirus type 2. PV3 is poliovirus type 3.
