# Supplementary material for: Persistence of poliovirus types 2 and 3 in waste-impacted water and sediment
Source: PLoS One. 2022 Jan 26;17(1):e0262761. doi: 10.1371/journal.pone.0262761 (PMC8791527; doi:10.1371/journal.pone.0262761)
Supplement: S3 Table — (DOCX) [file pone.0262761.s004.docx]

**Table S1**: Best Fitting Models for each Dataset

| Dataset | **Model** | **k1** | **k2** | **k3** | **nRMSE** | **Adjusted R^2^** |
| --- | --- | --- | --- | --- | --- | --- |
| PV2, Liquid, Culture at 4°C | jm2 | -2.22 | 1.14 | NA | 0.10 | 0.88 |
| PV2, Liquid, Culture at 21°C | jm1 | 0.10 | 0.27 | NA | 0.05 | 0.96 |
| PV2, Liquid, Culture at 30°C | jm1 | 0.28 | 0.28 | NA | 0.05 | 0.96 |
| PV2, Liquid, qPCR at 4°C***** | jm1 | 0.00 | 0.17 | NA | 0.22 | 0.51 |
| PV2, Liquid, qPCR at 21°C | gzm | 4.26 | -3.25 | 8.80E-03 | 0.14 | 0.62 |
| PV2, Liquid, qPCR at 30°C | gzm | 26.82 | -17.18 | 7.00E-03 | 0.07 | 0.90 |
| PV2, Sediment, Culture at 4°C***** | jm1 | 0.00 | 0.291 | NA | 0.20 | 0.36 |
| PV2, Sediment, Culture at 21°C | ep | 0.07 | NA | NA | 0.04 | 0.98 |
| PV2, Sediment, Culture at 30°C | ep | 0.26 | NA | NA | 0.04 | 0.98 |
| PV2, Sediment, qPCR at 4°C | gzm | -0.61 | -0.28 | 0.05 | 0.06 | 0.93 |
| PV2, Sediment, qPCR at 21°C | gzm | 15.09 | -12.70 | 0.05 | 0.21 | 0.55 |
| PV2, Sediment, qPCR at 30°C***** | NA | NA | NA | NA | NA | NA |
| PV2, WWO, Culture at 4°C | gz | 0.08 | -0.02 | NA | 0.07 | 0.95 |
| PV2, WWO, Culture at 21°C | jm2 | -2.85 | 2.97 | NA | 0.00 | 1.00 |
| PV2, WWO, Culture at 30°C | jm1 | 0.95 | 1.57 | NA | 0.01 | 1.00 |
| PV2, WWO, qPCR at 4°C | ep | 0.02 | NA | NA | 0.28 | 0.36 |
| PV2, WWO, qPCR at 30°C***** | jm2 | 1.18 | 1.09 | NA | 0.18 | 0.72 |
| PV3, Liquid, Culture at 4°C | bi3 | 0.12 | 0.11 | 10.90 | 0.15 | 0.78 |
| PV3, Liquid, Culture at 21°C | dep | 0.79 | 0.05 | 0.93 | 0.05 | 0.96 |
| PV3, Liquid, Culture at 30°C | jm1 | 0.43 | 0.46 | NA | 0.04 | 0.99 |
| PV3, Liquid, qPCR at 4°C***** | jm2 | -0.36 | 0.66 | NA | 0.26 | 0.21 |
| PV3, Liquid, qPCR at 21°C | jm2 | 3.97 | 0.61 | NA | 0.18 | 0.49 |
| PV3, Liquid, qPCR at 30° | bi3 | 2.14 | 2.12 | 2.56 | 0.07 | 0.94 |
| PV3, Sediment, Culture at 4°C***** | ep | 0.02 | NA | NA | 0.23 | 0.38 |
| PV3, Sediment, Culture at 21°C | dep | 15.73 | 0.08 | 0.49 | 0.07 | 0.94 |
| PV3, Sediment, Culture at 30°C | dep | 12.92 | 0.22 | 0.64 | 0.04 | 0.98 |
| PV3, Sediment, qPCR at 4°C | NA | NA | NA | NA | NA | NA |
| PV3, Sediment, qPCR at 21°C | jm2 | -220.55 | 46.62 | NA | 0.08 | 0.92 |
| PV3, Sediment, qPCR at 30°C | NA | NA | NA | NA | NA | NA |
| PV3, WWO, Culture at 4°C | gz3 | -2.61 | 0.06 | -0.89 | 0.08 | 0.91 |
| PV3, WWO, Culture at 21°C | ep | 0.71 | NA | NA | 0.08 | 0.92 |
| PV3, WWO, Culture at 30°C | jm1 | 0.53 | 0.06 | NA | 0.03 | 0.98 |
| PV3, WWO, qPCR at 4°C***** | ep | 0.03 | NA | NA | 0.21 | 0.62 |
| PV3, WWO, qPCR at 21°C | jm2 | -8.66 | 5.44 | NA | 0.14 | 0.84 |
| PV3, WWO, qPCR at 30°C | jm2 | 0.16 | 1.80 | NA | 0.09 | 0.88 |

^a.^ Model did not provide a good fit to the data (nRMSE≥0.20).
